# Dietary patterns, food groups and nutrients in Crohn’s disease: associations with gut and systemic inflammation

**DOI:** 10.1038/s41598-020-80924-8

**Published:** 2021-01-18

**Authors:** S. Ali Naqvi, Lorian M. Taylor, Remo Panaccione, Subrata Ghosh, Herman W. Barkema, Naomi Hotte, Nusrat Shommu, Sandeep Kaur, Raylene A. Reimer, Karen L. Madsen, Maitreyi Raman

**Affiliations:** 1 Department of Community Health Sciences and Department of Production Animal Health, Cumming School of Medicine and Faculty of Veterinary Medicine, University of Calgary, Calgary, AB T2N 4N1 Canada; 2 Department of Medicine, University of Calgary, Room 6D60, TRW Building, 3280 Hospital Drive NW, Calgary, AB T2N 4N1 Canada; 3 Department of Medicine, University of Alberta, Edmonton, T6G 2E1 AB Canada; 4 Faculty of Kinesiology, University of Calgary, Calgary, AB T2N 4N1 Canada; 5 Present Address: NIHR Biomedical Research Centre, Institute of Translational Medicine, University of Birmingham and Birmingham University Hospitals, Birmingham, B15 2TT UK

**Keywords:** Diseases, Gastroenterology, Medical research

## Abstract

This study examined associations between dietary intake and gut and systemic inflammation assessed by fecal calprotectin ≤ or > 100 μg/mg (FCP), C-reactive protein ≤ or > 5 mg/L (CRP) and serum cytokine profiles in Crohn’s disease (CD) patients in clinical remission. A 3-month observational study was conducted at the University of Calgary in Calgary, Alberta, Canada between 2016 and 2018 in 66 outpatients with CD in clinical remission. FCP was obtained from stool samples at baseline and 3-months and serum CRP and serum cytokines were assessed at 3-months only (n = 41). Dietary intakes were collected using 3-day food records at baseline and 3-months and categorized as: PREDIMED Mediterranean diet scores (pMDS) total and individual components, the dietary inflammatory index (DII), food groups, and common micro- and macro-nutrients. Statistical models were developed to identify relationships between dietary factors and FCP, CRP and cytokine levels. Daily intake of leafy green vegetables was associated with FCP ≤ 100 μg/mg (*p* < 0.05). Increasing omega 6:3 ratio was associated with CRP ≤ 5 mg/L (*p* = 0.02). Different cytokines were significantly associated with various dietary variables. Future studies in patients with greater disease activity should be undertaken to explore these relationships.

## Introduction

Inflammatory bowel disease (IBD) is believed to arise from a shared interaction between genetic and environmental influences^1^, resulting in an imbalance between pro- and anti-inflammatory signalling. There has been a steady rise in incident cases of IBD over the past several decades with the highest prevalence rates seen in Europe and North America of approximately 0.3% of the population^2^. Its recent increase in incidence across newly industrialized countries suggests environmental factors induce or modify disease expression. This has occurred in parallel with global dietary shifts towards a more “Western” style of eating. Common medical therapies include corticosteroids, immunomodulatory and biologic agents, and surgery. However, these therapies are not universally effective, the disease may recur and drugs may be associated with serious side effects. Recently, guidelines have included dietary therapies as a treatment option^3^.

Western diets are characterized by high intakes of total, polyunsaturated fatty acids (PUFA) and saturated fats, red and processed meats, commercial baked goods, sweetened beverages and food additives such as carrageenan, maltodextrin and emulsifiers together with low intakes of phytochemicals, fibre, fruits, vegetables and fish^4–8^. This dietary pattern can alter the gut microbiota and impair intestinal barrier function, thus resulting in a cascade of reactions that may trigger an enhanced inflammatory response. In contrast, prospective cohort and intervention studies have identified certain dietary patterns and individual diet components that may reduce gut inflammation and improve symptoms in IBD^7,9–12^. While knowledge of dietary relationships with the gut microbiome in IBD is evolving, the effects of dietary patterns and components on systemic markers of immune function is unexplored.

Given this context, it is justified to investigate the effect of dietary patterns and individual dietary components on surrogate biomarkers of Crohn’s disease (CD) activity and systemic markers of inflammation. Fecal calprotectin (FCP) is a useful surrogate marker of gastrointestinal inflammation and levels less than 100 µg/g are consistently associated with disease remission, whereas levels > 250 µg/g predict disease relapse^13^. C-Reactive Protein (CRP) is a marker of systemic inflammation and is associated with therapeutic response in IBD, and CRP levels correlate modestly with endoscopic disease activity^14^. Furthermore, serum cytokines have been associated with clinical remission and mucosal healing. With the above described rationale for these disease specific biomarkers, our study objective was to examine associations between gut and systemic inflammation assessed FCP ≤ or > 100 μg/mg, CRP ≤ or > 5 mg/L with dietary intake in patients with CD in remission. Exploratory analyses between serum cytokine profiles and dietary components were undertaken to characterize the relationship between diet and systemic markers of immune status.

## Material and methods

### Study design

A 3-month observational study of patients with CD was conducted at the gastrointestinal outpatient clinic at the University of Calgary, Alberta, Canada between 2016 and 2018.

### Inclusion/exclusion criteria

Inclusion criteria were: (a) ≥ 18 years of age; (b) documented diagnosis of luminal ileal and/or ileocolonic CD based on conventional criteria; (c) steroid-free clinical remission defined by a Harvey Bradshaw Index (HBI) < 5 for at least three months; d) achieved induction of remission either through anti-tumor necrosis factor (anti-TNF) agents or corticosteroids, and maintenance of remission with anti-TNF agents either with or without immunomodulators; and (e) able to provide informed consent, and read and write in English. Patients were excluded if they had (a) > 1 bowel resection; (b) evidence of fistulizing or stricturing disease CD; (c) upper gastrointestinal disease, or; (d) an ostomy.

### Data collection

Patient-related characteristics (i.e., age, sex, height, weight, body mass index: BMI) and medical history (i.e., treatment with anti-tumor necrosis factor biologics and immunomodulators at baseline and previous small bowel resection) were captured during the first study visit (n = 66). Stool was obtained at baseline and 3-months for FCP, whereas serum was collected for CRP and cytokine levels at 3 months only in patients who presented for timely follow-up collection (n = 41); patient demographics are presented in Table 1. Several inflammatory (Tumour Necrosis Factor alpha (TNF-α), interferon gamma (IFN-γ), interleukins 4, 6, 8, 17a, and 12/23p40 (i.e., IL-4, IL-6, IL-8, IL-17a, IL-12/23p40) and regulatory (IL-10 and IL-27) cytokines (all pg/mL) were examined. Cytokines were evaluated using electro-chemiluminescent, multi-plex ELISA kits by MesoScale Discovery (Rockville, MD, USA) as per the manufacturer’s protocol.

**Table 1:** Patient demographics and health characteristics at baseline and 3-month follow-up.

Variables	Baseline n = 66	Patients with cytokine measurement at time 2 n = 41	*P-*value of difference n = 41
Median age, years, (IQR)	51 (37–58)	53 (36–60)	–
Gender, males (%)	32 (49)	18 (44)	–
Median disease duration, years (IQR)	10.0 (4.5–17.8)	10.5 (4.9–19.0)	–
Median body mass index, kg/m^2^, (IQR)	27.3 (23.5–29.7)	27.5 (24.8–29.5)	–
Current smoker, n (%)	3, (4.5)	1, (2.4)	–
Anti-TNF use, n (%)	53 (80)	29 (71)	–
Steroid use, n (%)	0	0	–
Immunomodulators use, n (%)	29 (44)	16 (39)	–
Surgical Hx, n (%)	18 (27)	8 (20)	–
Median dietary inflammatory index, energy adjusted, (IQR)	+ 1.9 (0.5–2.8)	+ 2.2 (1.1–3.2)	0.06^a^
Median Mediterranean diet score, (IQR)	4.0 (4.0–5.0)	4.0 (3.0–5.0)	0.08^a^
**Fecal calprotectin**			
μg/mg Median (IQR)	73 (28–216)	73 (25–288)	0.79^a^
Biological remission,	42 (64)	22 (54)	0.18^b^
≤ 100 μg/mg (%)			
**C-reactive protein mg/L**			
Median (IQR)	n/a	1.47 (0.80–3.79)	n/a
≤ 5 mg/L		9 (22)	
TNF-α, pg/mL, median (IQR)	n/a	1.10 (0.77–1.44)	n/a
IFN-γ, pg/mL, median (IQR)	n/a	2.87 (1.90–5.10)	n/a
IL-4, pg/mL, median (IQR)	n/a	0.024 (0.02–0.04)	n/a
IL-6, pg/mL, median (IQR)	n/a	0.28 (0.17–0.39)	n/a
Il-8, pg/mL, median (IQR)	n/a	6.02 (5.43–7.62)	n/a
IL-17A, pg/mL, median (IQR)	n/a	1.70 (1.23–2.47)	n/a
IL-12/23p40, pg/mL, median (IQR)	n/a	75.12 (49.08–101.35)	n/a
IL-10, pg/mL, median (IQR)	n/a	0.16 (0.12–0.29)	n/a
IL-27, pg/mL, median (IQR)	n/a	370.66 (286.45–472.59)	n/a

^a^*P*-value computed using the Wilcoxon Signed-Rank test for paired data.

^b^*P*-value computed using McNemar’s Chi-squared test for paired data.

Dietary intake was determined using 3-day weighed food records at baseline and 3-months. Study participants met with the study coordinator to receive training on how to record an accurate 3-day weighed food record and were instructed to document all food and drinks consumed and preparation method on two representative non-consecutive weekdays, and one weekend day. The study dietitian reviewed the food record with the patient to identify any missing information.

Dietary intake data was analyzed using ESHA Food Analysis Software Version 11.3 X, ESHA Food Processor Nutrition Analysis Software (Salem, OR, USA), and individual macro- and micro-nutrients amounts were calculated. Dietary intakes were categorized using the following:

1. Mediterranean Diet Score (pMDS^15^), modified to exclude red wine consumption^8^, was calculated for each patient to measure how closely their overall dietary pattern resembled a Mediterranean diet.
2. The dietary inflammatory index (DII) developed by Shivappa et al.^16^ was calculated to score the inflammatory potential of individual dietary patterns.
3. Canada’s Food Guide to Healthy Eating (CFGHE) food group serving sizes was used to examine servings of fruits, vegetables, leafy green vegetables, red and processed meats, legumes, fish, and nuts and seeds^17^. Definitions of serving sizes for each food group have been previously reported^8^.

### Statistical analyses

All data management and statistical analyses were conducted in R version 3.5.3^18^, and a *p-*value < 0.05 was considered statistically significant. Outcomes of interest included FCP, dichotomized at 100 μg/mg to discriminate between patients in active or inactive intestinal inflammation^19^; CRP dichotomized at 5 mg/L^20^, and various inflammatory and/or regulatory cytokines (Table 1). Dietary variables explored in this analysis included total pMDS and its sub-components^8^; the DII, CFGHE food group servings; macro- and micro-nutrient intake and miscellaneous aspects of dietary intake (Tables 2, 3). Other patient characteristics including age in years at baseline, body mass index, previous bowel surgery, height, sex, taking anti-TNF medication or immunosuppressants, and weight were assessed as potential confounders of the associations of interest.

**Table 2:** Description of dietary pattern variables in the mixed-stepwise selection process for the regression models.

Variables	Units/levels	Median (IQR) at baseline (n = 66)	Median (IQR) at follow-up (n = 41)
**Diet scores**			
Mediterranean diet score (pMDS)	–	4.00 (4.00–5.00)	4.00 (3.00–5.00)
Dietary inflammatory index (DII), energy adjusted	–	1.89 (0.46–2.78)	2.16 (1.10–3.27)
**Food groups**			
Vegetables 1/2 cup, 1 medium or 1 cup leafy greens	Servings/d	1.30 (0.58–2.43)	1.42 (0.61–3.03)
Leafy green vegetables, 1 cup raw	Servings/d	0.30 (0.00–0.80)	0.25 (0.00–1.02)
Fruit, 1/2 cup or 1 medium and juice, 1/2 cup	Servings/d	1.60 (1.00–2.78)	1.33 (0.67–2.70)
Fruit, 1/2 cup or 1 medium, no juice	Servings/d	1.50 (0.78–2.63)	1.28 (0.67–2.38)
Legumes, ¾ cup	Servings/wk	0.00 (0.00–0.00)	0.00 (0.00–0.00)
Nuts, ¼ cup	Servings/wk	0.25 (0.00–4.40)	1.17 (0.00–2.63)
Red and processed meat, 84 g (3 oz)	Servings/d	1.15 (0.40–1.90)	0.33 (0.00–0.69)
Red meat, 84 g (3 oz)	Servings/d	0.40 (0.00–1.13)	0.00 (0.00–0.47)
Processed meat, 84 g (3 oz)	Servings/d	0.40 (0.08–0.93)	0.50 (0.00–0.43)
Fish and shellfish (84 g)	Servings/wk	0.00 (0.00–4.10)	2.33 (0.00–8.11)
Fish (84 g)	Servings/wk	0.00 (0.00–2.60)	1.05 (0.00–7.06)

**Table 3:** Description of macro- and micronutrients and single dietary components included in a mixed stepwise collection for regression models.

Variable	Type	Units/levels	Median (IQR) at baseline (n = 66)	Median (IQR) at follow-up (n = 41)
**Macronutrients**				
Calories	Measured	kcal	2123.21 (1635.79–2401.25)	1838.41 (1611.78–2372.05)
Carbohydrates	Measured	g	248.49(192.85–287.67)	227.88 (186.52–274.27)
Fiber	Measured	g	20.09 (15.07–29.72)	19.72 (16.20–28.23)
Fat	Measured	g	80.09 (53.97–99.67)	66.00 (56.84–97.07)
Mono-unsaturated fatty acids	Measured	g	15.27 (10.11–24.77)	13.97 (7.27–20.67)
Omega-3	Measured	g	0.71 (0.48–1.15)	0.74 (0.37–1.01)
Omega-6	Measured	g	6.00 (3.81- 10.02)	4.94 (2.44–7.12)
Omega6:Omega3	Continuous; computed	–	8.74 (6.05–10.75)	7.31 (4.12–9.71)
Poly-unsaturated fatty acids	Measured	g	8.84 (5.69–12.52)	7.20 (4.11–9.14)
Saturated fat	Measured	g	25.13 (17.28–32.41)	23.96 (16.90–34.80)
Trans-fats	Measured	g	0.55 (0.18–1.20)	0.56 (0.20–0.81)
Cholesterol	Measured	mg	280.90 (204.36–400.24)	226.17 (141.91–376.47)
Protein	Measured	g	89.66 (70.16–108.73)	82.39 (61.97–102.00)
**Single dietary components**				
Vitamin A	Measured	μg	439.58 (264.85–711.58)	410.10 (231.01–702.14)
Beta-carotene	Measured	μg	712.29 (202.36–2962.85)	680.41 (194.79–2061.24))
Vitamin B1	Measured	mg	1.05 (0.68–1.46)	0.69 (0.47–1.08)
Vitamin B2	Measured	mg	1.33 (0.99–2.04)	1.09 (0.68–1.70)
Vitamin B3	Measured	mg	16.60 (11.83–22.80)	13.06 (8.97–14.51)
Vitamin B6	Measured	mg	86.85(66.21–121.99)	1.09 (0.69–1.80)
Folate, B9	Measured	μg	263.03 (158.77–353.83)	162.23 (117.27–251.73)
Vitamin B12	Measured	μg	2.54 (1.95–4.48)	2.58 (1.52–3.92)
Vitamin C	Measured	mg	66.17 (49.83–130.92)	71.44 (39.91–127.54)
Vitamin D	Measured	μg	2.01 (1.10–3.47)	2.40 (1.09–4.24)
Vitamin E	Measured	mg	3.76 (2.76–6.70)	2.74 (2.02–4.25)
Iron	Measured	mg	13.06 (10.40–16.09)	10.80 (9.42- 15.92)
Magnesium	Measured	mg	188.32 (141.63–279.11)	161.99 (110.13–233.85)
Potassium	Measured	mg	1894.46 (1494.45–2542.69)	1755.77 (1094.58–2282.07)
Selenium	Measured	μg	72.73 (45.85–104.94)	52.54 (37.50–74.47)
Sodium	Measured	mg	3316.86 (2503.30–4239.60)	2720.15 (2150.07–3497.78)
Zinc	Measured	mg	5.70 (4.48–9.83)	5.06 (3.04–6.62)
Alcohol/d	Measured	g	0.00 (0.00–6.47)	0.00 (0.00–5.21)
Caffeine	Measured	mg	94.68 (18.97–189.44)	76.98 (10.03–217.01)

The association between FCP and diet was assessed using a mixed-effects logistic regression model to account for within-patient correlations between baseline and follow-up, and was implemented using the R package “lme4”^21^. This allowed the model to account for the within-patient correlation affecting the strength of the associations as it incorporated a random-effects term in the model to capture this correlation. Linear regression models were used to determine the association between diet and cytokine measurements using the R “stats” package (R Core Team, 2019), while logistic regression models were used to determine the association between CRP and diet.

Descriptive statistics and visualization were used to identify and explore outliers. The “ggplot2” library was used for all exploratory visualizations and plotting. Regression models were then built in a mixed stepwise manner, beginning with a forward-stepwise selection based on univariable associations with the outcomes (*P* < 0.15), followed by a backwards-elimination while assessing confounding (removal of the variable causes a change in the remaining coefficient estimates of ± 30%), and retained only the variables with *P* < 0.10. Interactions between variables were not assessed due to the small sample sizes resulting from the stratification necessary to test the corresponding interactions.

### Ethical approval

The study was approved by the Calgary Health Research Ethics Board (REB15-1805) and informed consent was provided by all patients. All the research was carried out in accordance with relevant guidelines and regulations.

## Results

### Demographics

Table 1 describes the demographics and health characteristics for patients at baseline and 3-months. At baseline, participants were generally middle aged (Median = 51 years, IQR = 37–58) overweight (Median = 27.3 kg/m^2^, IQR = 23.5–29.7) and 49% were male. All patients were in clinical remission (HBI < 5) and 64% of patients were in biological remission defined as FCP ≤ 100 μg/mg. At baseline, 53 patients (80%) were receiving anti-TNF biologics, with 29 patients (44%) receiving immunomodulators and no patients receiving corticosteroids.

### Dietary pattern and macro- and micro-nutrients

Patients’ DII scores were generally pro-inflammatory (Mean = 1.6, SD = 1.8) and pMDS scores showed only 4–5 healthy behaviours being performed (Mean = 4.6, SD = 1.5) out of a possible 13. Means and proportions of dietary patterns, individual food groups and macro- and micro-nutrients used in the analyses are described in Tables 2 and 3.

### Fecal calprotectin

**Figure 1:**
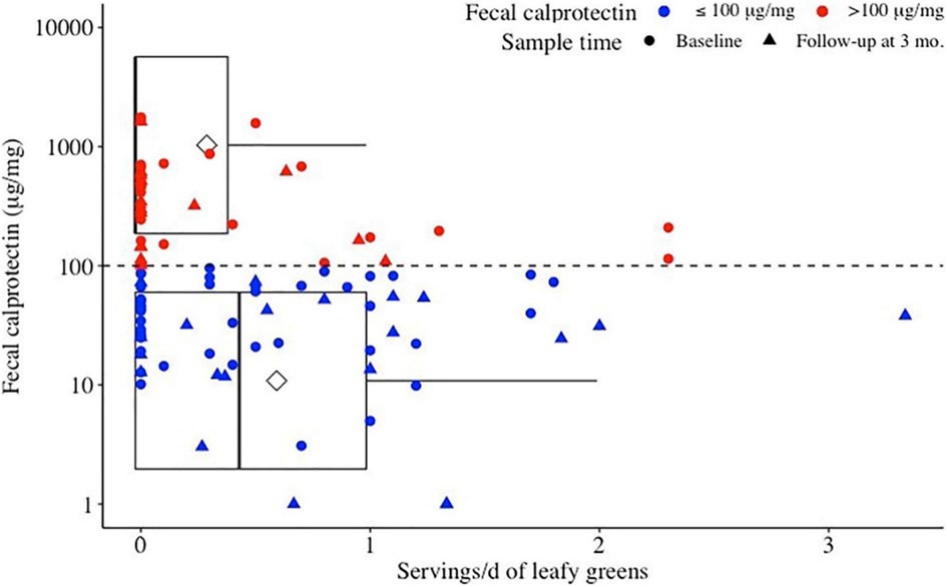
Fecal calprotectin ≤ 100 μg/mg is significantly related to leafy greens intake.

Increasing daily servings of leafy green vegetables were associated with FCP ≤ 100 μg/mg (*p* < 0.05; Table 4, Fig. 1).

**Table 4:** Final multivariate models for fecal calprotectin and C-reactive protein, and pro- and anti-inflammatory cytokines.

Outcome	Variable	Coefficient (95% CI)	Standard error	*P*-value
**Fecal calprotectin**				
> 100 μg/mg	Reference^a^	1.04 (− 0.40 to 3.20)	0.79	0.19
	Leafy green vegetables/d	− 1.14 (− 2.64 to − 0.12)	0.58	< 0.01
	Vitamin B2 intake	− 0.90 (− 2.48 to − 0.03)	0.54	0.09
**C-reactive protein**				
> 5 mg/L	Reference	1.95 (− 0.14 to 4.61)	1.17	0.10
	Omega 6:3 ratio	− 0.37 (− 0.76 to − 0.10)	0.16	0.02
	Sex	− 2.91 (− 6.20 to -0.68)	1.33	0.03
**Pro-inflammatory cytokines (pg/ml)**				
TNF-α	Reference	− 0.46 (− 1.45 to 0.53)	0.487	0.35
	Age	0.01 (− 1.92 × 10^–3^ to 0.01)	0.01	0.10
	Body mass index	0.04 (3.15 × 10^–3^ to 0.08)	0.02	0.03
IFN-γ	Reference	1.17 (− 2.33 to 4.66)	1.72	0.50
	Processed meat consumption	17.06 (9.83 to 24.29)	3.57	< 0.01
IL-4	Reference	4.71 × 10^–3^ (− 0.01 to 0.02)	0.01	0.40
	Alcohol consumption	− 5.81 × 10^–4^ (− 9.89 × 10^–4^ to − 1.72 × 10^–4^)	2.30 × 10^–4^	0.01
	Not taking anti-TNF	8.61 × 10^–3^ (− 3.95 × 10^–4^ to 0.02)	4.42 × 10^–3^	0.06
	Iron intake	1.68 × 10^–3^ (8.03 × 10^–4^ to 2.55 × 10^–3^)	4.12 × 10^–4^	< 0.01
IL-6	Reference	0.02 (0.22 to 0.18)	0.10	0.85
	Fiber intake	0.01 (1.69 × 10^–3^ to 0.02)	0.01	0.02
	Weekly fish consumption	− 0.01 (− 0.03 to 2.90 × 10^–3^)	0.01	0.10
	Omega-3 fatty acids	0.37 (0.18 to 0.55)	0.09	< 0.01
	Poly-unsaturated fatty acids	− 0.02 (− 0.05 to 3.95 × 10^–3^)	0.01	0.10
IL-8	Reference	2.05 (− 0.41 to 4.51)	1.20	0.10
	Alcohol consumption	− 0.09 (− 0.15 to − 0.02)	0.03	0.01
	Beta-carotene intake	4.11 × 10^–4^ (1.74 × 10^–5^ to 8.05 × 10^–4^)	2.23 × 10^–4^	0.04
	Fiber intake	0.09 (0.01 to 0.17)	0.04	0.03
	Inadequate fruit pMDS^b^	1.89 (0.41 to 3.37)	0.72	0.01
	Vitamin B3 Intake	0.07 (0.01 to 0.16)	0.04	0.07
IL-17A	Reference	1.80 (0.66 to 2.96)	0.57	< 0.01
	Inadequate fruit pMDS^b^	− 0.77 (− 0.15 to − 0.09)	0.34	0.03
	Not taking anti-TNF medication	− 0.70 (− 1.30 to − 0.09)	0.30	0.03
	Total pMDS^b^	0.21 (0.03 to 0.39)	0.09	0.03
Il-12/23 p40	Reference	− 86.31 (− 200.16 to 27.54)	55.96	0.13
	Body mass index	7.26 (3.37 to 11.14)	1.91	< 0.01
	Energy adjusted DII^c^	− 10.34 (− 20.07 to − 0.61)	4.78	0.04
**Anti-inflammatory/regulatory cytokines**				
IL-10	Reference	0.11 (− 0.04 to 0.25)	0.01	0.14
	Beta-carotene intake	1.06 × 10^–4^ (4.65 × 10^–5^ to 1.65 × 10^–4^)	3.20 × 10^–5^	< 0.01
IL-27	Reference	45.70 (− 135.52 to 226.91)	89.17	0.61
	Leafy green vegetables/day	− 75.14 (− 123.18 to − 27.11)	23.6	< 0.01
	Red meat/day	− 78.23 (− 135.11 to − 21.36)	27.99	< 0.01
	Weight (kg)	1.06 (0.57 to 1.56)	0.24	< 0.01

^a^Reference groups of subjects refer to theoretical individuals where their observed value for all variables in the model is 0 for continuous variables. When sex is included in the model, the reference group is females.

^b^Modified Mediterranean Diet Score (pMDS), modified to exclude red wine consumption was calculated for each patient to determine how closely their overall dietary pattern resembled a Mediterranean diet.

^c^Dietary inflammatory index.

### C-reactive protein

**Figure 2:**
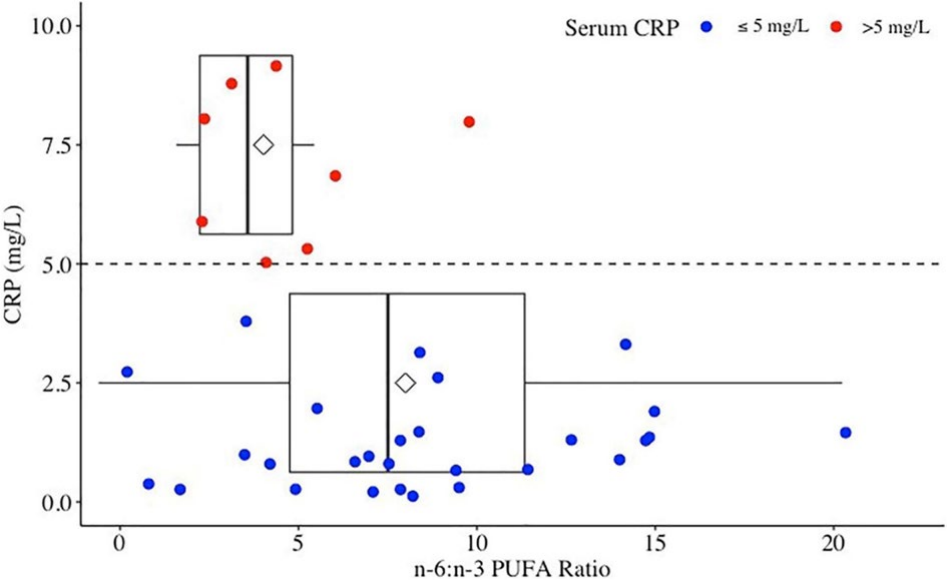
C-reactive protein ≤ 5 mg/L is significantly related to ratio of omega-6 to omega-3 polyunsaturated fat.

When assessing the odds of having a low CRP at 3-months (defined as a serum concentration ≤ 5 mg/L), males had higher odds of having a low CRP than females (Table 4). In addition, a higher omega 6:3 fatty acids ratio increased the odds CRP would be ≤ 5 mg/L (Fig. 2). The mean omega 6:omega-3 (n-6:n-3) PUFA ratio in the low CRP group was 8:1, compared to the high CRP group with a ratio of 4:1.

### Serum cytokines

Serum cytokine relationships with dietary variables are presented in Table 4. Different cytokines were significantly associated with various dietary variables. Considering dietary pattern, a negative association between energy adjusted DII and IL-12/23 p40 (*P* = 0.04), and positive association between pMDS and IL-17A (*P* = 0.03) was observed, pMDS and DII scores were not associated with the remaining serum cytokines.

## Discussion

We present the first prospective study to characterize the relationship between pMDS, DII, CFGHE food groups, micro- and macro-nutrients with serum and fecal biomarkers of disease activity and peripheral cytokine profiles in patients with CD in clinical remission. Daily servings of leafy green vegetables was associated with FCP ≤ 100ug/mg, and an n-6:n-3 PUFA ratio of 8:1 was associated with CRP ≤ 5 mg/L. We did not identify relationships for FCP or CRP with dietary patterns measured by pMDS and energy-adjusted DII.

Epidemiological studies have associated fruit, vegetable and fish intake with lower risk of IBD incidence^22,23^. Leafy greens are a rich source of carotenoids which may help improve intestinal barrier function and reverse lipopolysaccharides induced intestinal barrier damage through enhancing the expression of tight junction proteins^24^. A plant-based diet, rich in dietary fiber, appears to be beneficial for human health by promoting the development of a diverse microbial ecosystem^25^; in this study however, total dietary fiber was not associated with FCP or CRP. Exclusive enteral nutrition (EEN), a low-fibre product, is used effectively as primary therapy for CD in pediatric patients with luminal CD^26^. EEN may deplete the nutrients (e.g., fibre) necessary for bacterial growth, or create a luminal microenvironment where the functionality of inflammatory microbes is suppressed, inhibiting the gut mediated immune response in patients with CD^27^. Therefore, the mechanisms underlying the protective benefits of leafy greens may extend beyond their fiber content.

Higher n-6:n-3 PUFA ratios were associated with increased odds of patients having a CRP ≤ 5 mg/L. In typical Western diets this ratio can be as high as 20:1^28^, and disproportionate n-6:n-3 intake ratios may lead toward a proinflammatory response which may affect the onset of IBD^29^. Supplementation with n-3 PUFA to alter the n6:n3 ratio towards anti-inflammatory effects has shown mixed results in CD and genetic polymorphisms in n-3 metabolism could be one possible mechanism to explain conflicting results^29^. A study in zebrafish compared various levels of n-6:n-3 ratios in fish consuming a standard diet of 12% total calories from lipids^30^. Similar to our study, this study identified an 8:1 ratio as optimal compared to a 5:1 and a 2:1 ratio to promote greater gains in lean body mass and less total body fat. In addition, zebrafish eating the n-6:n-3 PUFA 8:1 ratio diet had lower levels of CRP and amyloid, markers of inflammation identified on liver biopsies. The authors concluded the ratio of n6:n3 may influence the inflammatory response less than the total percent of calories consumed from lipids. In our study, total intake of fat grams did not have any significant association with FCP, CRP or inflammatory cytokines.

Having identified dietary associations with FCP and CRP, we proceeded to examine potential immune mediated mechanisms to explain these results. Both expected and unexpected relationships between peripheral cytokines and dietary components and patterns were identified.

Unexpectedly, increasing scores for the pMDS (more anti-inflammatory dietary pattern) were associated with increasing levels of IL-17A. Similarly, lower energy adjusted DII scores (more anti-inflammatory) were associated with increased IL12/23p40. In 320 healthy Iranian women, an inverse relationship with IL-17A and adherence to a Mediterranean diet was identified^31^ while another study demonstrated increasing consumption of fruit juices reduced IL-17A^32^. Although IL-17A has generally been classified as a pro-inflammatory cytokine, it may regulate components of the intestinal barrier by reinforcing tight junction proteins in epithelial cells, and it induces the expression of mucin-associated genes and the production of β-defensins in the colon^33,34^. IL-17A may have protective effects and its role in gut homeostasis may not be completely understood.

In previous work the DII score was associated with increased levels of TNF-α and IFN-γ and not associated with IL12/23p40 as seen in this study^35^; however, this was in healthy adolescents and not in a diseased cohort of patients. In another study in adults, the DII was positively associated with IL-6 and TNF-α^36^. The DII was not significantly associated to these cytokines in our study; however, our study collected data using 3-day food records compared to a food frequency questionnaire, did not control for the same confounders as the previous work, and the study populations were different.

We identified a significant relationship between IL-10 and β-carotene intake. IL-10 is an immunoregulatory cytokine that plays a crucial role in orchestrating intestinal immune homeostasis^37^. However, these data need to be interpreted with caution due to the relatively small sample size and the possible disconnect between serum cytokine levels and gut inflammation. β-carotene is widely distributed in green leafy, yellow and orange coloured vegetables and fruits, and is one of the most abundant carotenoids. β-carotene has been reported to suppress LPS-induced inflammatory responses by inhibiting nuclear factor kappa B expression, suggesting an anti-inflammatory role^38^.

We demonstrated an inverse relationship between red meat intake and IL-27 serum levels, and an inverse relationship between leafy green vegetables and IL-27. It has not been definitively determined whether IL-27 ameliorates or promotes intestinal inflammation, as contradictory roles for IL-27 have been reported in animal models of IBD. Studies have demonstrated the ability of IL-27 to lessen colonic inflammation by increasing the production of IL-10 in animal models of colitis^39,40^. These somewhat conflicting results may speak to the dual effects of IL-27 on intestinal inflammation, however mechanistic interpretations cannot be concluded at this time.

Study strengths include dietary data collection and FCP measurements at two time points using dietitian reviewed 3-day food records, and rigorous statistical modeling. Study limitations to the regression-based model analysis were related largely to sample size. We selected inclusion and exclusion criteria that would allow us to have a clinical population that was representative of the typical patient in remission without being too restrictive or broad. All patients presenting to the outpatient clinic were informed of the study and over the 2-year period we were able to achieve an initial n = 66. This sample size allowed us sufficient power to examine our question but certainly suggests that future larger studies are warranted to further confirm the interesting findings of our study.”As the number of variables and dietary factors observed were greater than the number of individuals, a risk factor analysis was underpowered to begin with. This small sample size made it impossible to assess interactions between predictor variables in the models, meaning that some key relationships between variables may not have been identified, as effect modification was not tested. As the purpose of this work was exploratory to provide preliminary associations for further investigation, reducing the number of significant associations detected by adjusting the significance level may result in potential relationships not being further investigated. In addition, while serum cytokine levels do not necessarily reflect mucosal inflammation, the identified relationships may add context and foundation for future dietary mechanistic studies. Raw *P*-values have been included in the results tables so they can be interpreted independently of a pre-defined significance cutoff.

In conclusion, we have identified relationships between dietary components and FCP and CRP in patients with CD in clinical remission, and provided preliminary associations between dietary components, patterns and nutrients with serum cytokines. The lack of robust associations between the DII and pMDS with FCP and CRP were unanticipated; however, the remitted patient population may limit these associations, and future studies building on this work should be undertaken in patients with greater disease activity.
